# PLA/PHB-Based Materials Fully Biodegradable under Both Industrial and Home-Composting Conditions

**DOI:** 10.3390/polym14194113

**Published:** 2022-09-30

**Authors:** Mária Fogašová, Silvestr Figalla, Lucia Danišová, Elena Medlenová, Slávka Hlaváčiková, Zuzana Vanovčanová, Leona Omaníková, Andrej Baco, Vojtech Horváth, Mária Mikolajová, Jozef Feranc, Ján Bočkaj, Roderik Plavec, Pavol Alexy, Martina Repiská, Radek Přikryl, Soňa Kontárová, Anna Báreková, Martina Sláviková, Marek Koutný, Ahmad Fayyazbakhsh, Markéta Kadlečková

**Affiliations:** 1 Institute of Natural and Synthetic Polymers, Faculty of Chemical and Food Technology, Slovak University of Technology, Radlinského 9, 812 37 Bratislava, Slovak Republic; 2 Institute of Materials Science, Faculty of Chemistry, Brno University of Technology, Purkyňova 464/118, 612 00 Brno, Czech Republic; 3 Department of Landscape Engineering, Hortyculture and Landscape Engineering Faculty, Slovak University of Agriculture, Hospodárska 7, 949 76 Nitra, Slovak Republic; 4 Department of Environmental Protection Engineering, Faculty of Technology, Tomas Bata University in Zlín, Nad Ovčírnou III 3685, 760 01 Zlín, Czech Republic

**Keywords:** polylactic acid (PLA), polyhydroxybutyrate (PHB), blend polymeric material, biodegradation, industrial compost, home-compost

## Abstract

In order to make bioplastics accessible for a wider spectrum of applications, ready-to-use plastic material formulations should be available with tailored properties. Ideally, these kinds of materials should also be “home-compostable” to simplify their organic recycling. Therefore, materials based on PLA (polylactid acid) and PHB (polyhydroxybutyrate) blends are presented which contain suitable additives, and some of them contain also thermoplastic starch as a filler, which decreases the price of the final compound. They are intended for various applications, as documented by products made out of them. The produced materials are fully biodegradable under industrial composting conditions. Surprisingly, some of the materials, even those which contain more PLA than PHB, are also fully biodegradable under home-composting conditions within a period of about six months. Experiments made under laboratory conditions were supported with data obtained from a kitchen waste pilot composter and from municipal composting plant experiments. Material properties, environmental conditions, and microbiology data were recorded during some of these experiments to document the biodegradation process and changes on the surface and inside the materials on a molecular level.

## 1. Introduction

Biodegradable polymers are one of the possible alternatives to conventional polymeric materials that can, for specific applications, provide the benefit of biological decomposition without leaving unwanted litter or potentially dangerous microplastics [1,2]. However, biodegradable polymers and materials based on them are significantly different in the conditions needed for their biodegradation and in the time frame of their biodegradation [3,4,5]. Ideally, for a given application, we need a material that fulfils the necessary application properties and most important mechanical properties and at the same time can biodegrade under the conditions related to the particular application. It is often difficult to find or develop a material that fulfils all of those requirements.

Mainly with the aim to achieve suitable processing and mechanical and barrier properties for a given application, real polymer materials are often blends of several polymers and contain various additives and fillers [6,7,8]. The whole material has to be biodegradable, which means that correct selection of individual components is very important and can significantly affect its biodegradability. The plasticizer triacetin, used in a study by Sedničková et al. [9], was tested with PLA and PLA/PHB blends, and the materials were exposed to biodegradation in compost at 58 °C. The results of the study prove higher sensitivity of PHB (polyhydroxybutyrate) towards biodegradation in comparison with that of PLA under the same conditions. Additionally, the plasticizer triacetin degraded faster in comparison with PLA. The study also showed that changes in material composition (e.g., amount of plasticizer) might change the biodegradation rate. Another bio-based plasticizer, acetyl-tri-n-butyl citrate (ATBC), in combination with polyethylene glycol (PEG) was also tested as a component of PLA/PHB blends [10]. The material exhibits disintegration under composting conditions in less than one month. The ability of PHB to act as a nucleating agent in PLA/PHB blends slowed down the disintegration, while plasticizer content accelerated it. 

It is expected in many cases that waste containing biodegradable plastics is collected along with other primarily plant-based organic waste and it is further treated in municipal or agricultural composting plants. In general, materials suitable for such an end-of-life should comply with EN 13432 standard describing procedures for testing under so-called industrial composting conditions [11]. According to this standard, industrial composting requires an elevated temperature (55–60 °C) in combination with relatively high water activity expressed as water content (approximately 60% *w*/*w*) and the presence of oxygen. Under such conditions, several criteria must be met: (i) The disintegration of the material from at least 90% must take place within 12 weeks, (ii) 90% mineralization of the composted material must be achieved in less than six months, which is usually measured from evolved CO_2_, and (iii) the material should not have a negative effect on compost quality (no or minimal heavy metal content, no ecotoxicity) [12].

The description above, however, does not correspond to the conditions on a typical simple composting plant where temperatures over 50 °C can be achieved for about two to three weeks maximally [13]. Then, the process continues in a milder mesophilic temperature range. To address this issue, a group of standards was formulated describing so-called “home-composting” (Vicotte OK compost HOME, AS 5810 Austrian standard) [14]. Here, besides other requirements, the temperature should be kept between 20 and 30 °C during the biodegradation test. Despite the fact that some commercially used materials declared as compostable polymers are often used in applications where composting is meant to be the final stage of life of these materials, they do not meet these requirements and therefore are not compostable. Chemical and biological processes, in general, are, to some extent, accelerated with temperature [15,16]. For example, PLA needs temperatures well over 50 °C to initiate the biodegradation process, which is possibly related to the glass transition temperature of PLA occurring in approximately the same temperature range [17]. This fact was demonstrated, e.g., by Sedničková et al. [9] in a study, where the biodegradation of PLA was measured in compost incubations at 25, 37, and 58 °C for 119 days. The mineralization achieved at 58 °C was 92.3%, but only 19.5% at 37 °C and 14.9% at 25 °C. Other studies showed no or limited PLA biodegradation under mesophilic conditions [18,19]. 

Moreover, in reality, the almost complete mineralization must be achieved within six months, most of the time at relatively mild temperatures, to make the material compatible with the typical composting plant operation settings [12]. As a consequence, today, typical relatively simple composting plants with no fundamental process control possibilities often tend to reject biodegradable polymer materials, even those labelled as compostable, because, according to their experiences, these items do not decompose fast enough, complicate operations at the plant, and contaminate the resulting compost [20,21].

Another prospective material, PHB, also produced from renewable resources [22,23], can, in contrast to PLA, reach 100% mineralization in five weeks under mild conditions, e.g., in the soil [24]. This polymer itself does not have sufficient processing and mechanical properties for many applications.

The main aim of this study is to demonstrate the biodegradation of the original bio-based biodegradable materials and some model products made of these materials under conditions corresponding to industrial composting (58 °C) and some of them even under conditions corresponding to home-composting (28 °C) in a reasonable time frame. The samples were also evaluated in a real municipal composting plant. Other supporting methods were performed to follow the biodegradation process. This study is meant to show that the presented materials with very good mechanical and processing properties are also biodegradable and fully compatible with common composting technology in a simple municipal composting plant or even in the proper home-compost.

## 2. Materials and Methods

### 2.1. Materials 

Samples made of PLA/PHB or PLA/PHB/TPS (thermoplastic starch) blends for this study were developed for various processing technologies and applications. Thermoplastic starch TPS was prepared by blending corn starch + glycerol 70/30 in a twin screw extruder at 160 °C. The used plasticizers are based on esters of citric acid. Blends according to composition in Table 1 were prepared by blending in a co-rotating twin screw extruder with the following parameters: L/D = 44, D = 26 mm, temperature profile from hopper to head: 50-160-170-170-170-170-170-170-160-160 °C, screw speed 150 rpm. The blends were cooled in a water bath and pelletized. All blends were sent to production companies that made the test products. The basic characteristics of all the used blends are listed (Table 1).

The test specimens based on PLA/PHB or PLA/PHB/TPS are listed in Table 2. Cups were produced by the company KS-PT s.r.o. (Slovakia), the thermoforming sheet was produced in Panara a.s. (Slovakia), and blown films were produced in Topstav s.r.o. (Slovakia) in their pilot (Panara a.s.) plant or production plant (KMS-PT s.r.o. and Topstav s.r.o.). The smaller picture of the sample represents the specific used test specimen for biodegradation testing in home-compost, industrial compost, and an electric composter. 

### 2.2. Biodegradation Testing by CO_2_ Production Quantification

Composting biodegradation tests were performed according to the adapted and miniaturized ISO 14855 method in 500 mL biometric flasks with septum-equipped stoppers. Mature compost from a nearby municipal composting facility (TSZ Ltd., Zlín, Czech Republic) was used in this part of the study. This test was done at 58 °C for industrial composting and at 28 °C to simulate home-composting conditions. Into each flask, 2.5 g of dry-weight compost, 5 g of perlite, and 1 mL of mineral salt medium were weighed, and the water content of the substrate mixture was eventually adjusted to 60% by the addition of sterile drinking water. One hundred milligrams of the samples were cut into 5 × 5 mm fragments that were placed in each sample flask. For each sample, three flasks plus 4 blank flasks were used. The internal production of CO_2_ in blank incubations was always subtracted to calculate the net sample mineralization. Headspace gas was sampled at appropriate intervals through the septum with a gas-tight needle and conducted through a capillary into a gas analyzer (UAG, Stanford Instruments, Sunnyvale, CA, USA) to determine the amount of CO_2_. Biodegradation percentage (D_t_) was calculated as

(1) $$D_{t}=\frac{{\left({\mathrm{CO}}_{2}\right)}_{t}-{\left({\mathrm{CO}}_{2}\right)}_{b}}{{\mathrm{ThCO}}_{2}}\times 10$$

where (CO_2_)_t_ is the released CO_2_ by each sample, (CO_2_)_b_ is the CO_2_ produced by the blank flasks, and (ThCO_2_) is the theoretical CO_2_ from the sample. A flash elemental analyzer 1112 (Thermo Fisher Scientific, Waltham, MA, USA) was used to measure the carbon content of the samples.

### 2.3. Compostability Testing in an Electric Composter

A small electric composter GG 02 from the JRK company (Slovakia) was used for biodegradation testing while the samples were incubated together with kitchen waste. The effective volume of the composter was 40–50 liters. The temperature was 65 °C during the whole operation time, except for one hour per day when the temperature increased to 75 °C to ensure the hygienization of the content. The internal stirrer was activated for 20 min during each hour, providing altered mixing sequences in forward and reverse directions. The biodegradation process was initiated according to the user manual using the original ACIDULO^®^ bacteria culture. Samples of PLA/PHB or PLA/PHB/TPS blends were inserted into the composter two weeks after the stabilization of the process in the composter. Each day, 0.5–1.0 kg of kitchen food waste was added to the composter. The samples were weighed before being inserting into the composter. Only one sample was measured for every composition because of technical reasons of the experiments. The content of the composter was removed each week and sieved through a sieve with a mesh size of 2 × 2 mm. Pieces larger than 2 mm (which did not pass through the sieve) were collected from the fraction above the sieve. The collected samples were washed in water, subsequently dried in an air oven for 1 h at 90 °C, and weighed with precision of 0.0001 g. Then, the samples were returned to the composter immediately after weighing. Biodegradation was evaluated as the percentage of disintegration. Microbiology inside the composter was monitored with DNA isolation and sequencing following an already established methodology [25].

### 2.4. Disintegration Testing in a Municipal Composting Plant

**Figure 1:**
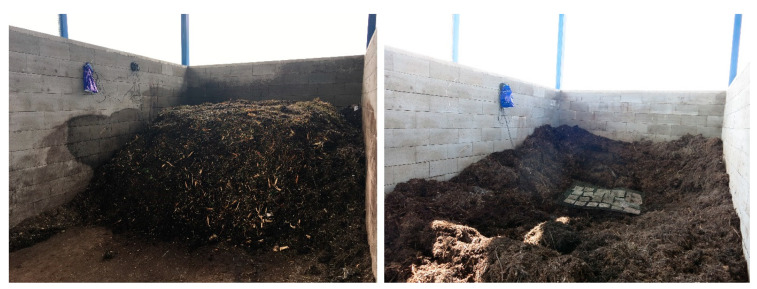
The compost pile and the plastic net with the cut samples.

The disintegration of samples was tested also in the municipal composting facility of the city of Nitra (Nitra District, Southwest Slovakia) under real conditions of industrial composting. The compost pile consisted of approximately 11 m^3^ of biodegradable municipal waste (a family house garden and public greenery plant-based waste). Disintegration testing was realized in two independent 12-week-long composting cycles. The first cycle was realized from 6 July 2019 to 27 September 2019, and the second cycle—from 16 July 2020 to 12 October 2020 on a dedicated roofed site. Both cycles followed a certified methodology [26]. The samples were weighed and enclosed in a plastic net with a 2 × 2 mm mesh diameter. The cut samples were inserted to approximately 2/3 of the height of the compost pile (Figure 1).

Right below the samples, probes were placed to measure the temperature and humidity inside the compost pile. The data were continuously monitored and recorded. The samples were inspected every 2–3 weeks; sample packages were carefully removed from the pile, visually inspected, and photographed. Afterwards, they were again inserted to 2/3 of the height of the compost pile. The residues of the samples were dried and weighed at the end of the cycle. Biodegradation was evaluated as the percentage of disintegration. The outside air temperature (in both cycles) was about 22 °C on average. 

During the first cycle, only one specimen was tested from each sample. The average value of humidity during the entire monitored period was 39.3% (vol). The average value of inner temperature during the first cycle was 62.6 °C. During the second cycle, two specimens were tested for each sample. The average humidity during the entire monitored period was 35% vol. The average inner temperature during the second cycle was 61.1 °C. 

### 2.5. Material Characterization Methods

**SEM microscopy.** Surface changes on the tested films were observed using SEM. The samples were coated by a gold/platinum alloy using Balzers SCD 050 sputtering equipment. TESLA BS 300 was used for the observation of samples composted in an electric composter, and JEOL F 7500 SEM (JEOL, Tokio, Japan) was used for the samples from an industrial city composting plant. Phenom Pro Desktop SEM (Thermo Fisher Scientific, Waltham, MA, USA) was used for laboratory experiments under industrial and home-composting conditions.

**Thermophysical properties’ measurement.** Differential scanning calorimetry (DSC) was used for the determination of basic thermophysical properties such as glass transition temperature, crystallization temperature, and melting temperature of samples after 0, 6, and 13 days of composting. The conditions for DSC measurements are in Table 3. 

**Gel permeation chromatography(GPC) measurements.** Samples (5 mg) were dissolved in chloroform (1 mL) at 70 °C and filtered through 0.45 µm polytetrafluoroethylene (PTFE) syringe filters. GPC was performed in a 185 Agilent HPLC series 1100 chromatograph (Santa Clara, CA 95051, United States) with a PLgel mixed-c 5 μm, 7.5 × 300 mm column, with chloroform as the mobile phase. Twelve polystyrene standards (0.2–2000 kDa) were used for calibration.

## 3. Results and Discussion

### 3.1. Characterization of the Studied Materials

PLA/PHB- and PLA/PHB/TPS-based materials and final products (films, sheets, and cups) are described in Table 1 and Table 2. The materials were developed to be compostable in industrial compost or even under home-composting conditions while still having favorable processing and service properties and containing an important proportion of PLA, which is probably the most available bio-based polymer but still considered to be non-biodegradable under home-composting conditions. All specimens listed in Table 2 were tested in the electric composter, municipal composting plant, and laboratory tests under industrial composting conditions. Samples in the film form (D, E, F) were also tested for biodegradability in a laboratory test under home-composting conditions. The selection of these samples for home-composting was based on their relatively low thickness and on the assumption that home-composting conditions are less aggressive than those in industrial composting, especially for the PLA component of the materials [27,28].

### 3.2. Biodegradability in the Laboratory Test under Industrial and Home-Composting Conditions 

All samples were exposed to the laboratory test under industrial composting conditions (58 °C). It was expected that based on the composition of the samples; they all should be completely mineralized under these conditions. For the thick-wall samples without TPS (A and B), more time to reach 100% mineralization was assumed.

**Figure 2:**
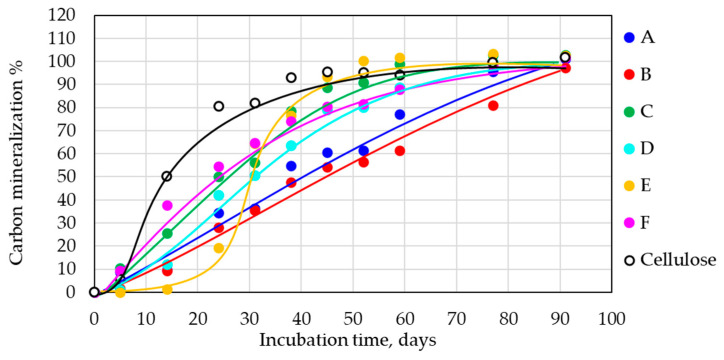
Mineralization of samples under industrial composting conditions, 58 °C.

Three samples for each composition were measured, including the reference sample (cellulose). The average standard deviation for all tested compositions was ±8.6 for the industrial composting conditions. Mineralization of 100% was obtained for all tested samples after about 90 days of incubation (Figure 2). No sample except E exhibited a lag phase. Sample E contained the highest amount of PLA (70%), so this typical feature of PLA compost biodegradation was demonstrated in this sample [28,29]. All other samples, which degraded without a lag phase, contained at least 30% of easily biodegradable PHB and/or TPS, which were able to smooth out the lag phase in the biodegradation curve under industrial compost conditions. In the case of E, the PHB phase was probably closed inside the dominant PLA phase. After the lag phase, mineralization went on exponentially, and the sample reached complete mineralization as the first one. The mineralization was also fast for other film samples D and F, with F being faster at the beginning, which probably reflected its higher content of easily biodegradable TPS and plasticizers (40%) in comparison to that in D (5%). Surprisingly, the mineralization was also fast for a relatively thick (1 mm) sample C with high PHB and TPS contents (65%). The slowest degradation was observed in the case of both thick samples A (4 mm) and B (1 mm). TPS-containing cups exhibited slightly faster biodegradation in very good correlation with the cup’s construction (thickness). Interestingly, the thickness of the sample, at least to some extent, did not play a very significant role. The thin film F composed of a PLA/PHB/TPS blend exhibited a course of biodegradation similar to that of the thick sample C. Both were made of a comparable formulation, but if TPS was not present in the composition, thickness played a more important role (samples B, D, and E were TPS-free formulations). Possibly, the TPS phase could initiate early disintegration of the sample and thus circumvent the importance of thickness.

**Figure 3:**
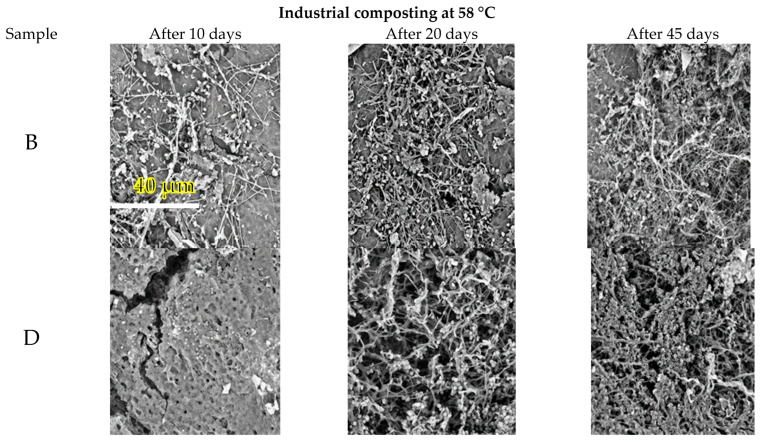

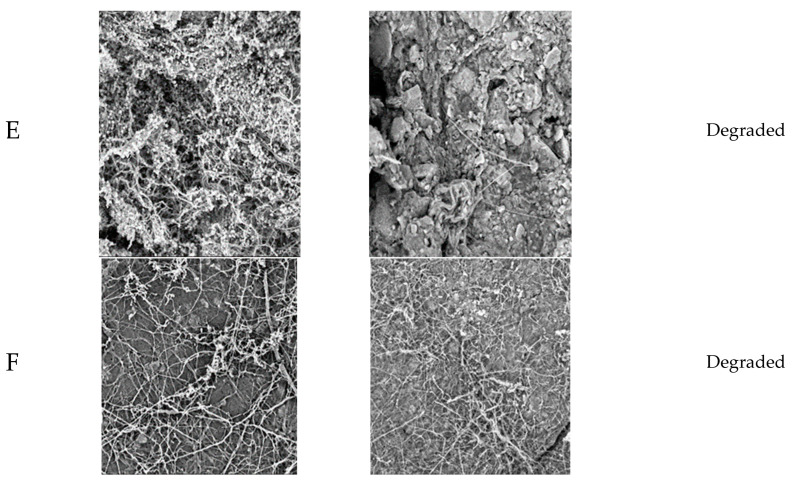
Scanning electron microscopy survey of the sample surface during the biodegradation test at 58 °C (industrial composting condition). Magnification of 5000×. Specifications according to Table 2 (B—500 mL cup, D—sheet for thermoforming, E—blown film and F—blown film with TPS).

The selected samples were removed from the compost after incubation, and they were observed in SEM to evaluate microbial colonization and deterioration of the material’s surface (Figure 3). After only 10 days in the compost, all materials were densely colonized, and the density of the biofilm was clearly increased with the time of exposure. It was not possible to identify the microorganisms present, but the morphological appearance suggested filamentous thermophilic actinobacteria with distinguishable round endospores. The erosion of the surface was also clearly apparent.

**Figure 4:**
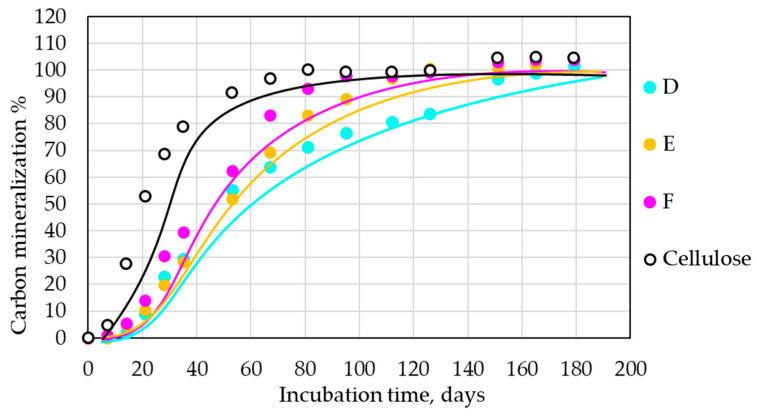
Mineralization of samples under home-composting conditions, 28 °C.

Film samples D, E, and F were selected for biodegradation under home-composting conditions (28 °C, Figure 4). From our previous experiences [28,29] and the majority of the scientific literature on the topic [30], it should be expected that the PLA fraction of the materials should not be mineralized under such conditions.

Three samples for each composition were measured, including the reference sample (cellulose). All samples exhibited total mineralization in a period of about 180 days. The average standard deviation for all tested compositions was ±3.9 for the home-composting conditions. An about 15-day-long lag phase occurred for all tested samples. Additionally, the curves for the tested samples were not so far from that of the cellulose reference and had the expected order. The thin film of the PLA/PHB/TPS blend (F) exhibited the fastest biodegradability, followed with a minimal gap by the thin film sample without TPS (E). In this case, apparently, thickness and plasticizer played a significant role; the thick film made of PLA/PHB without TPS and at lower plasticizer content (D) degraded significantly slower than did the thin film with higher plasticizer content (E).

**Figure 5:**
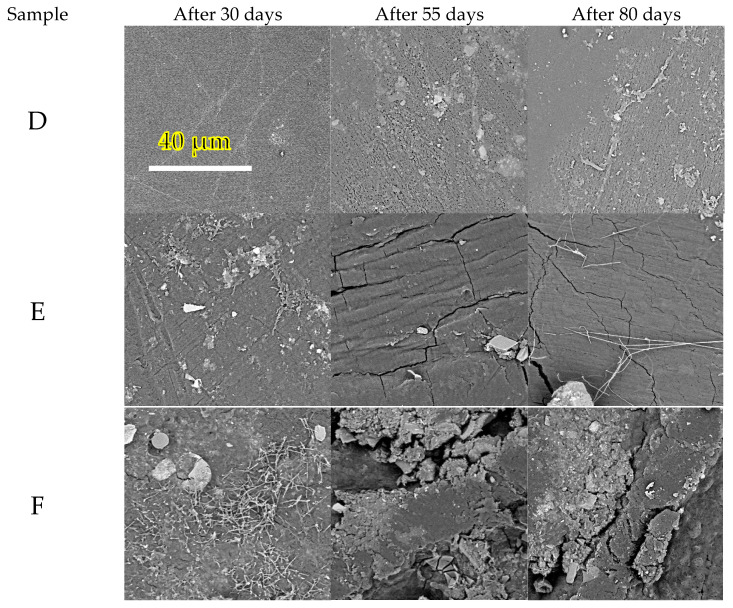
Scanning electron microscopy survey of the sample surface during the biodegradation test at 28 °C (home-composting conditions). Magnification of 5000×. Specifications according to Table 2 (D—sheet for thermoforming, E—blown film and F—blown film with TPS).

When observed in SEM (Figure 5), the samples from the home-compost experiments, in general, showed a much lower degree of surface colonization if compared with that in the industrial compost experiment, which can be explained by the fact that at this temperature, a completely different microbial community was present. Again, filamentous (actinomyces most probably) but also rod-shaped bacteria were discernible (e.g., Figure 5F, 30 days). (1) (2) (3) Cavities and cracks were gradually formed. At this temperature, fungi were very active, with their extracellular enzymes probably degrading the material even if they were not seen attached to the surface.

A very important result from the environmental as well as practical points of view is the fact that PLA/PHB-blend samples degraded fully at home-compost conditions. It means that 100% mineralization was reached despite the general opinion that PLA is not able to biodegrade at temperatures below its T_g_, (about 55 °C) under home-compost conditions and therefore only the mineralization of the non-PLA part of the blends could be expected. The blending of PLA and PHB in the hot melt state probably causes re-esterification reactions, leading to the formation of PLA/PHB co-polyesters. The easily biodegradable PHB segments in such a co-polyester can promote a release of low MW fragments that are prone to further biodegradation [27]. It is very difficult to investigate the occurrence and extent of re-esterification by standard analytical methods, but the results from the home-composting biodegradation can be considered as proof. This is indeed a significant discovery not only for the eventual compositing “at home” but also for industrial composting plants. In these usually municipal facilities, the composting process is quite simple and not always fully controlled, so the thermophilic phase of the composting process could be too brief or not sufficiently hot to initiate total PLA mineralization. Problems have been reported with various items from PLA-based material labelled as “compostable”, and often, such items are no longer accepted in these plants. Additionally, this will prevent microplastic formation, one of the most-closely watched environmental risks studied recently.

### 3.3. Composting in the Electric Composter

**Figure 6:**
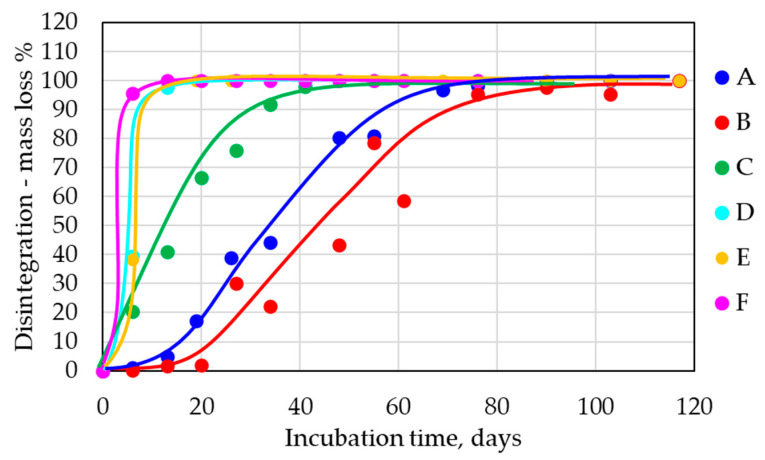
The disintegration of tested samples in a small electric composter, expressed as weight loss.

All samples in Table 2 were also tested in a small electric composter. Test specimens of 10 × 7 cm with the original thickness were inserted into the composter. Biodegradation was evaluated as weight losses in percentages; it can also be stated that the degree of disintegration was evaluated. The results presented in Figure 6 show differences between the disintegration of individual samples. The disintegration of all film samples was very fast, and all films were disintegrated into pieces smaller than 2 mm after 20 days. The cup containing TPS (C) exhibited fast disintegration into particles smaller than 2 mm after 40 days. The other two cups (B, PLA/PHB-based; A, TPS-containing cup) were disintegrated after a very similar time period, while the combined cup A was slightly faster. The comparison of biodegradation curves based on CO_2_ measurements (in industrial compost conditions and home-compost conditions (Figure 6) and disintegration curves (Figure 6) can provide an important insight into the problem of the eventual formation of microplastics.

**Figure 7:**
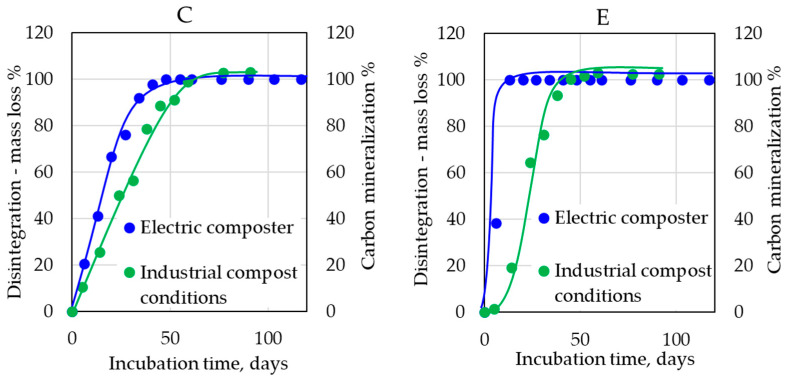
Comparison of biodegradation curves measured as CO_2_ in industrial compost conditions (58 °C) for samples C and E and in home-compost conditions (28 °C) for sample E with disintegration curves obtained using an electric composter for samples C and E.

In Figure 7, sample C is shown as a typical example of all three cups. It means that the composting process in this case ensured direct microbial conversion of the materials to CO_2_ with a relatively short disintegration step. On the contrary, the films rapidly disintegrated into smaller particles, and the mineralization followed immediately. This observation in the case of films was given by the low mechanical strength of the samples and the mechanical strain during the mixing inside the composter. A thick cup was more resistant to mechanical breakdown than thin films were before the samples became too brittle due to the biodegradation process. A similar effect was also observed when home-composting and electric composter biodegradation were compared in the case of sample E (Figure 7), where, logically, a more significant delay of mineralization after disintegration was detected. These results show that the studied materials really also underwent mineralization in home-composting conditions, and eventual fragments/microplastics after their disintegration were readily mineralized.

**Figure 8:**
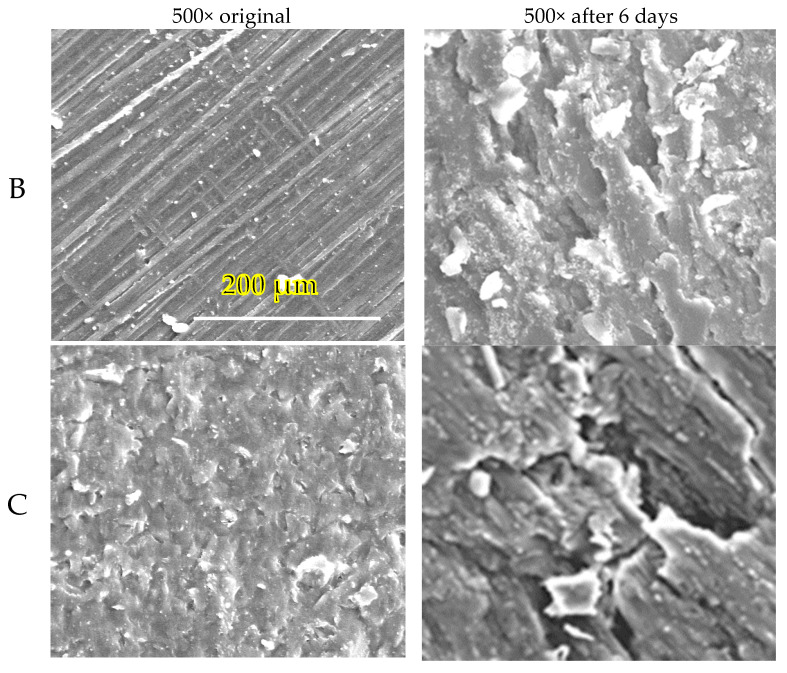
SEM images of the tested samples before composting and after 6 days of incubation in an electric composter. Specifications according to Table 2. (B—500 mL cup and C—200 mL cup with TPS).

All tested samples exhibited well-visible changes in the surface morphology after only 6 days of incubation, as can be seen in the SEM figures. Thick samples B without TPS and C with TPS that were not disintegrated instantly are shown as examples (Figure 8). The microscopic observation is in perfect correlation with the results of biodegradation and disintegration testing discussed previously. Cavities of various dimensions and depths were created in relation to the sample´s composition, which resulted in the enlargement of the surface area and acceleration of the biodegradation process.

**Figure 9:**
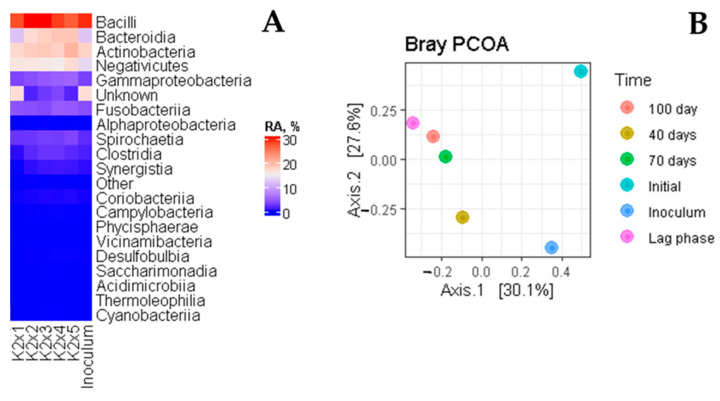
The bacterial community inside an electric composter. (**A**): heatmap at class taxonomic level, RA, relative abundance. (**B**): PCoA scatter plot.

The composition of the bacterial community was described by next-generation 16S r DNA metagenome sequencing in different stages of the process. The design of the composter and its operation exhibited a severe limitation for the bacterial community. Very high operation temperature (65 °C) and a daily hygienization period (75 °C) put even thermophilic bacteria on the limit of their survival. These parameters should be adjusted to provide a better environment for the compost microflora; however, it is not the topic of this study. Evidently (Figure 9), the community was dominated by thermophilic spore-forming taxa (Bacilli, Actinobacteria). The presence of other taxa like Bacteroidia, Negativicutes, and even Clostridia witness probably the presence of anaerobic pockets inside the compost. The introduced inoculum strongly influenced the initial stage, then, the gradual increase of the other taxa with time could be seen.

### 3.4. Composting at the Municipal Composting Plant

**Figure 10:**
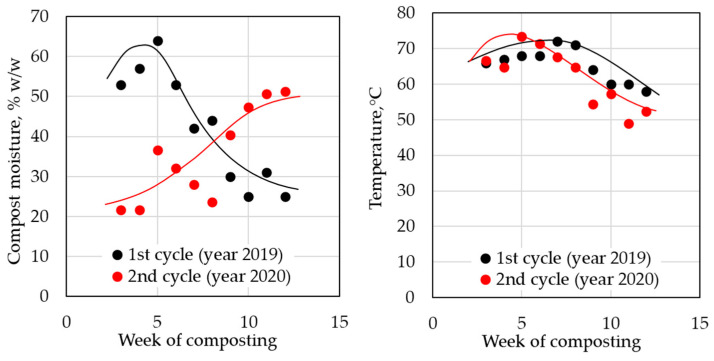
Moisture and temperature profiles during composting in a municipal composting plant.

The study of compostability under real conditions and verification of the results from the laboratory and the electric composter were realized in two composting cycles at Nitra municipal composting plant (Slovakia). Samples B, C, and F were tested during the first cycle. All investigated (Table 2) samples were tested in the second cycle. The first and second cycles were realized separately, the first one during the summer of 2019 and the second one during the summer of 2020. The main difference between the first and second cycles was in the humidity curve of the compost substrate (Figure 10). During the first cycle, the composting process started with a high level of moisture which then decreased over time. The moisture profile in the second cycle was exactly the opposite. In both cases, pure pulp paper (pure cellulose) was used as a reference. Biodegradation was evaluated from the weight losses after 12 weeks of incubation. 

**Figure 11:**
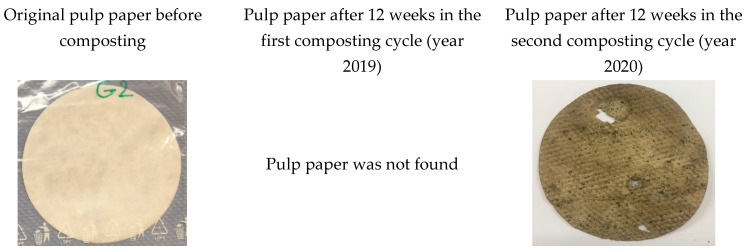
Pulp paper reference material before industrial composting and after 12 weeks of composting.

**Figure 12:**
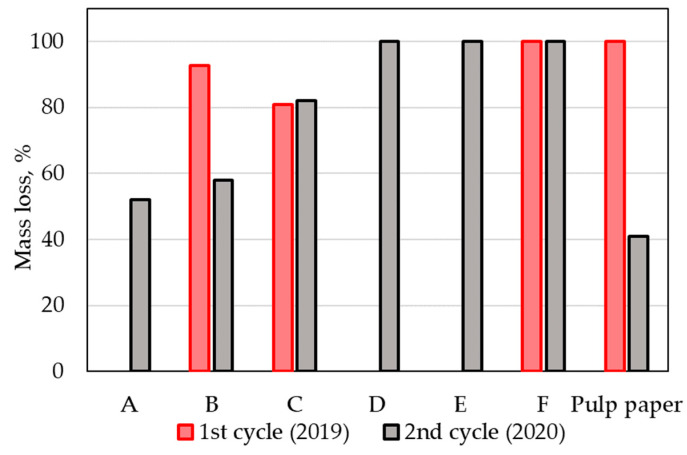
Weight losses of the tested samples after 12 weeks of incubation in the first and second composting cycles at a municipal composting plant.

Significant differences in the first cycle (year 2019) were observed in the degradation of the pulp paper reference in both cycles (Figure 11 and Figure 12). While temperature profiles were very similar in both cycles, the trends of the compost substrate humidity differed significantly. The first stages of composting in the second cycle proceeded apparently at insufficient humidity, which could explain why in the second cycle, the compost was not sufficiently active for the biodegradation of the reference material. Suitable moisture content is an essential parameter for the composting process, especially during the initial hot phases of the composting. The pulp paper reference was not found in the first cycle of composting after 12 weeks, while in the second cycle, with low humidity in the first stages of composting, lower than 50% weight loss was observed (Figure 12), and the paper still preserved its original shape (Figure 11).

The investigated samples provided very interesting results regarding the above-discussed differences between both composting cycles. Despite the humidity profile, film samples degraded completely in both composting cycles (sample F in the first composting cycle, as well as samples D, E, F in the second composting cycle). A thick cup sample containing TPS (sample C) degraded equally in both cycles, and the humidity profile had a small effect on their biodegradation. The thick sample without TPS (cup sample B) and the combined cup (sample A) were probably more sensitive to the moisture content but still biodegraded substantially. In general, the samples in the study biodegraded comparably or even better than the cellulose reference did in the described real-condition experiment. 

**Figure 13:**
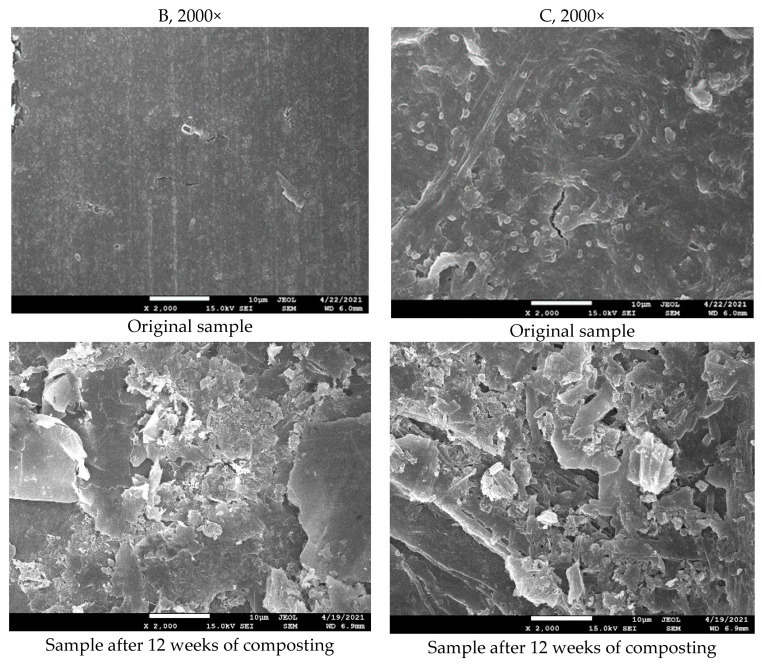
SEM images after 12 weeks of incubation at a municipal composting plant (second cycle).

The surface morphology of the samples was also studied in the second cycle of the industrial composting experiment. Film samples could not be analyzed because they were decomposed completely; all other samples exhibited significant changes in surface morphology (Figure 13). Similarly, as with the samples from the electric composter, strong surface erosion was observed.

### 3.5. Changes in the Material Properties during the Composting Experiments

**Figure 14:**
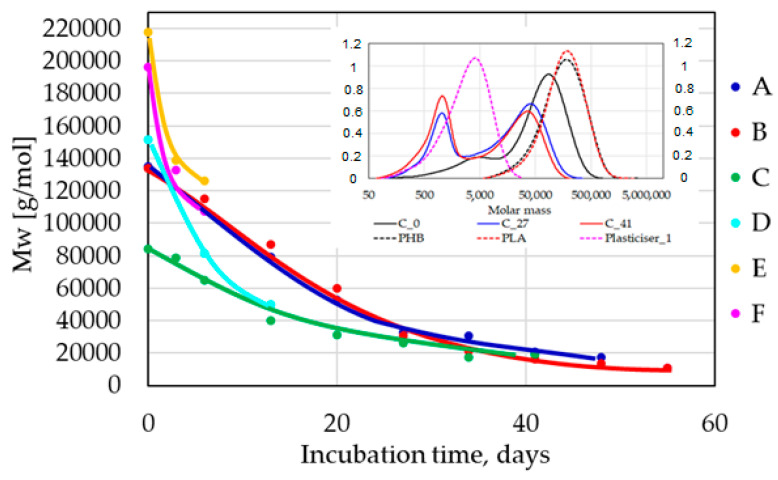
MW evolution during incubation in an electric composter. The GPC records of sample C after 0, 27, and 41 days of composting are shown as an example (insert).

In the case of the electric composter, after each period, molecular weight distributions were measured by GPC. It must be noted that GPC measurements were realized with solid, nondegraded residuals of composted materials. GPC curves of unprocessed PLA and PHB used in the blends were also analyzed, and the records are shown (Figure 13, insert). Partial degradation due to blending and then processing of the blend during sample preparation was noticeable for each sample. The degradation caused by processing only in the melt state was not very extensive; only in the case of sample C was its extent more significant. This can relate to the presence of glycerol in TPS and possible alcoholysis of PHB by glycerol [31,32]. The intense shift of the main peaks to lower molecular weights after composting was visible mainly in the case of thick cup samples. In addition, the appearance of low-molecular-weight fractions was clearly seen in GPC records. The last indicated fraction produced by the biodegradation process exhibited an average molecular weight (MW) of about 1000 g/mol. Lower MW fractions (below 500 g/mol) were probably easily mineralized. The fast gradual decrease of MW was observed for all samples (Figure 14).

Regardless of the original MW, all samples that did not biodegrade exhibited similar MW values after 30 days of composting. It means that the degradation process ran not only on the surface of the samples but also in the bulk of the materials. Only short time periods were evaluated in the case of film samples because already after 14 days of composting, no film sample could be retrieved.

**Figure 15:**
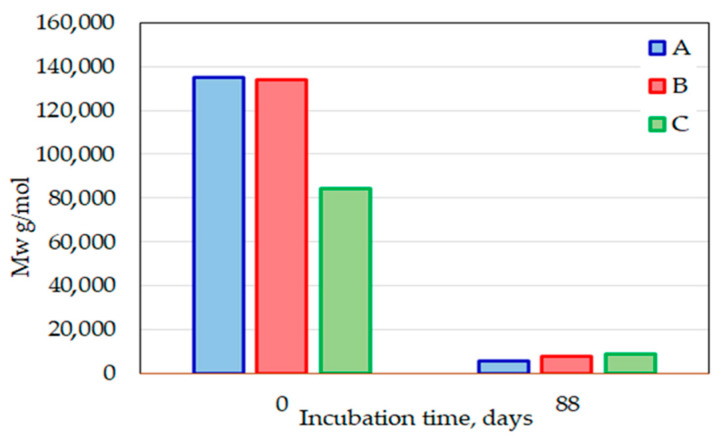
Comparison of MW for cup samples before composting and after 12 weeks in the second composting cycle (2020).

The process at the municipal composting plant also caused a dramatic decline in MW below 1000 g/mol, as shown for selected samples (Figure 15). Such low-molecular-weight substances are considered to be easily and rapidly biodegradable during the following composting period.

**Figure 16:**
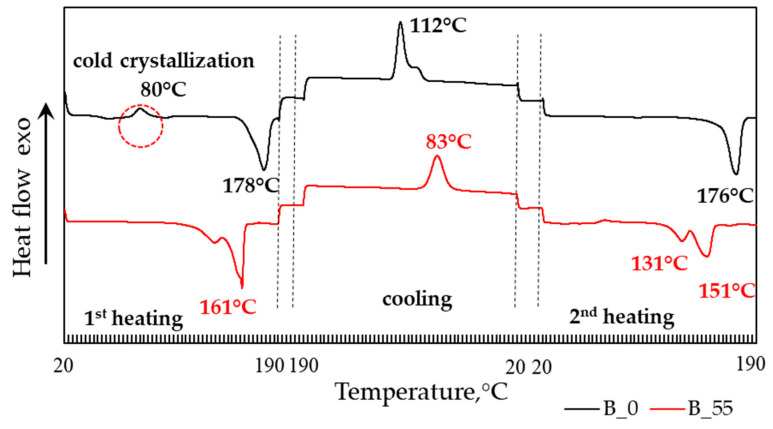
DSC curves of sample B before composting (B_0) and after 55 days of composting (B_55) in an electric composter.

The mentioned changes in polymer structure were also confirmed by DSC measurements realized on solid residuals after each evaluated period of composting process in the electric composter (Figure 16).

**Figure 17:**
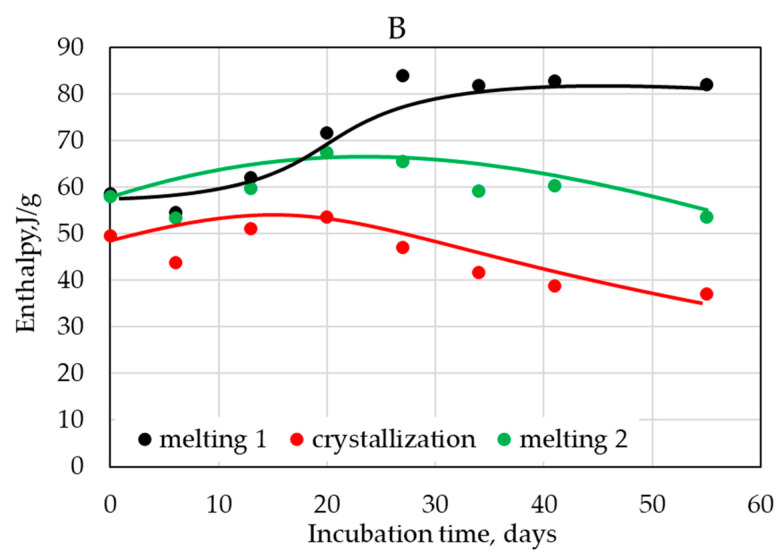
Evolution of enthalpies of melting and crystallization during the incubation in an electric composter for sample B.

Significant changes were visible in the first heating as well as in the cooling and the second heating cycle. The temperatures of melting were significantly shifted to lower values after 55 days of composting; after the same period, the crystallization temperature was significantly decreased. The composting caused the disappearance of the cold crystallization peak visible in the original sample, and the melting peak was split in two. Quantitative analysis of DSC measurements (Figure 17) also showed that the changes in thermograms in the first run were not connected only to the consumption of the amorphous phase, but significant changes in the molecular structure occurred. This was confirmed by the decrease of crystallization enthalpy as well as of the melting enthalpy in the second heating cycle when the record of the sample was no longer affected by its previous thermal and other history.

## 4. Conclusions

This detailed study presents results from composting experiments in several experimental settings, all aimed at the group of PLA/PHB blend materials with various compositions and, depending on the particular material, also containing citric-acid-ester-based plasticizers and thermoplastic starch (TPS). All tested samples were in their real final product forms and shapes.

It was found that the studied PLA/PHB-based blends were fully biodegradable under industrial composting conditions as well as in an electric composter, which was designed for the composting of kitchen waste. A very important result from this study is the observation that some of the studied materials, despite their high PLA contents, could fully biodegrade under home conditions. The result is explained by the assumption that PLA could react with PHB during the blending in the melt form, and this reactive extrusion process could induce re-esterification of both polymer components. This extrusion process was intentionally designed with such a purpose. The easily biodegradable PHB segments can promote the polymer chains’ scission and ultimately complete biodegradation of the materials under home-composting conditions. 

Evaluation of GPC, DSC, and SEM data showed that during the composting of studied PLA/PHB blends, the changes in the molecular structure and morphology proceeded not only on the surface but also in the bulk of the materials. SEM images showed that already in the first stages of biodegradation, not only macroscopic disintegration but also biological decomposition of the materials took place on the surface, causing an enlargement of the specific surface area and thus the acceleration of the biodegradation process. 

A very important result was the verification that disintegration and mineralization as the two main processes during biodegradation of materials could run in the case of the studied PLA/PHB blends not only in sequence but also in parallel. This was observed mainly in the case of thicker specimens like cups, but also in the case of thin products like films where the disintegration was extremely rapid but the mineralization phase was delayed only shortly. Based on these results, it can be concluded that these PLA/PHB materials do not leave microplastics in the environment after industrial as well as after home-composting processes. The composting experiment in a municipal composting plant confirmed and verified the laboratory results. During two independent testing cycles, it was found that the studied PLA/PHB materials degraded comparably or even faster than the pure cellulose reference did.

## Figures and Tables

**Table 1:** Basic characteristics of the tested materials.

Blend No.	Blend Technology	PLA Ingeo 4043D %wt.	PHB Enmat Y1000 %wt.	ATBC Citrofol B2 %wt.	TPS %wt.	MFI	Ρ	TS	ε	T_g_
IM 2	Injection moulding	40	50	10	0	5.8	1.2	31	8	N/A
IM 1	Injection moulding	30	40	5	25	35	1.3	34	5	53
TF -1	Thermoforming	65	30	5	0	6.2	1.2	28	36	N/A
FB 2	Film blowing	70	15	15	0	18	1.2	18	330	28
FB 1	Film blowing	50	10	15	25	33	1.3	11	288	24

MFI, melt flow index, 180 °C, 2.16 kg, g/10 min; Ρ, density g/cm^3^; TS, tensile strength at break, MPa; ε, elongation at break, %; T_g_, glass transition temperature, °C.

**Table 2:** Products that were tested for biodegradation and compostability.

Sample	Grade No	Thickness	Description
A, bicomponent cup 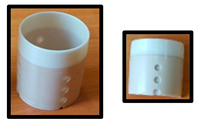	IM 2 (Inner layer)	1–6 mm, average 4 mm	Bicomponent cup
	IM 1 (Outer layer)		
B, 500 mL cup 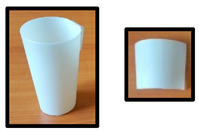	IM 2	1 mm	Cup for beverages and beer, mainly for festivals
C, 200 mL cup 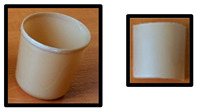	IM 1	1 mm	Cup for drinks suitable mainly for dining restaurants and canteens
D, sheet for thermoforming 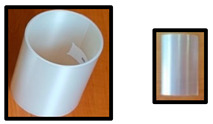	TF 1	0.35 mm	Semiproduct (sheet) for subsequent thermoforming technology, for various packaging applications
E, blown film 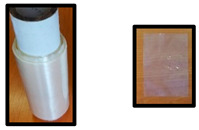	FB 2	0.04 mm	Blown film, e.g., for bags, suitable for various packaging applications
F, blown film 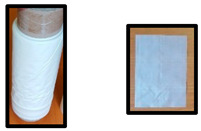	FB 1	0.04 mm	Blown film e.g., for bags, mainly for waste bags for biowaste collection

**Table 3:** Conditions for DSC measurements.

Phase	Ramp	Temperature, °C	Time, min
1. Conditioning	isothermal	0	1
2. Heating	10 °C/min	200	20
3. Conditioning	isothermal	200	1

## Data Availability

The study did not report any data.
